# Antimicrobial Biophotonic Treatment of Ampicillin-Resistant *Pseudomonas aeruginosa* with Hypericin and Ampicillin Cotreatment Followed by Orange Light

**DOI:** 10.3390/pharmaceutics11120641

**Published:** 2019-12-01

**Authors:** Seemi Tasnim Alam, Tram Anh Ngoc Le, Jin-Soo Park, Hak Cheol Kwon, Kyungsu Kang

**Affiliations:** 1 Natural Products Informatics Research Center, Gangneung Institute of Natural Products, Korea Institute of Science and Technology, Gangwon-do 25451, Korea; seemitasnim@kist.re.kr (S.T.A.); lntramanh@kist.re.kr (T.A.N.L.); jinsoopark@kist.re.kr (J.-S.P.); hkwon@kist.re.kr (H.C.K.); 2 Division of Bio-Medical Science & Technology, KIST School, Korea University of Science and Technology (UST), Gangwon-do 25451, Korea

**Keywords:** ampicillin, antimicrobial photodynamic therapy (APDT), *Caenorhabditis elegans*, hypericin, orange light, *Pseudomonas aeruginosa*

## Abstract

Bacterial antibiotic resistance is an alarming global issue that requires alternative antimicrobial methods to which there is no resistance. Antimicrobial photodynamic therapy (APDT) is a well-known method to combat this problem for many pathogens, especially Gram-positive bacteria and fungi. Hypericin and orange light APDT efficiently kill *Staphylococcus aureus*, methicillin-resistant *Staphylococcus aureus* (MRSA), and the yeast *Candida albicans*. Although Gram-positive bacteria and many fungi are readily killed with APDT, Gram-negative bacteria are difficult to kill due to their different cell wall structures. *Pseudomonas aeruginosa* is one of the most important opportunistic, life-threatening Gram-negative pathogens. However, it cannot be killed successfully by hypericin and orange light APDT. *P. aeruginosa* is ampicillin resistant, but we hypothesized that ampicillin could still damage the cell wall, which can promote photosensitizer uptake into Gram-negative cells. Using hypericin and ampicillin cotreatment followed by orange light, a significant reduction (3.4 log) in *P. aeruginosa* PAO1 was achieved. *P. aeruginosa* PAO1 inactivation and gut permeability improvement by APDT were successfully shown in a *Caenorhabditis elegans* model.

## 1. Introduction

The development of antibiotic resistance in many pathogenic microorganisms is a serious health problem worldwide, and alternative methods for treating multidrug-resistant microorganisms are urgently needed. The World Health Organization (WHO) published a report in April 2014 that declared that the “postantibiotic” era is approaching and that people will die because of minor infections or injuries because antibiotics will not be effective for treatment [1,2]. Strains of many Gram-positive and Gram-negative bacteria, such as *Staphylococcus aureus*, *Enterococcus faecium*, *Klebsiella pneumoniae*, *Acinetobacter baumannii*, *Pseudomonas aeruginosa*, and *Enterobacter* species, exhibit almost all types or classes of antibiotic resistance, and are commonly called “ESKAPE” pathogens, or superbugs [3]. Antimicrobial photodynamic therapy (APDT) is a reliable treatment that with the proper combination of photosensitive drugs and light, can effectively kill pathogenic bacteria. Moreover, APDT is an inexpensive and useful method where poor funding of health treatment is common [4]. Overall, APDT is promising because it can serve as an alternative to antibiotic treatment for many infections. PDT is a known therapy for treating various microbial species, viruses, fungi, inflammatory disorders, and cancers [5].

PDT relies on a photosensitive compound called a photosensitizer (Ps) [6]. The bacterial surface usually absorbs the Ps, and exposure to a specific wavelength of light triggered the excited singlet state (^1^Ps). Reactive oxygen species (ROS) or reactive molecules are produced by the excited electrons produced during transformation to a lower energy configuration, and the Ps is later converted to the triplet state (^3^Ps) [7]. There are two types of ROS generation mechanisms, type 1 and type 2 [7]. Type 1 reactions produce free radicals and radical anions or cations, such as O_2_^∙^^−^, H_2_O_2_, and ·OH; O_2_^∙^^−^ in particular can produce enough cytotoxic ROS, such as OH radicals, to oxidize biomolecules and cause cellular damage and even cell death. In type 2 reactions, a direct reaction occurs between the Ps and molecular oxygen (O_2_) to produce highly active ^1^O_2_ [7]. These reactions occur simultaneously in APDT but depend on the specific Ps applied and the environment during the chemical reactions [7]. In many cases, the type 2 (singlet oxygen) mechanism is primarily responsible for biological events, whereas the type 1 mechanism occurs in a low-oxygen or polar environment. ROS are produced by many mechanisms and oxidize various cellular compounds, for example, amino acids such as cysteine, methionine, tyrosine, histidine, and tryptophan as well as DNA, to cause cell death [7].

**Figure 1:**
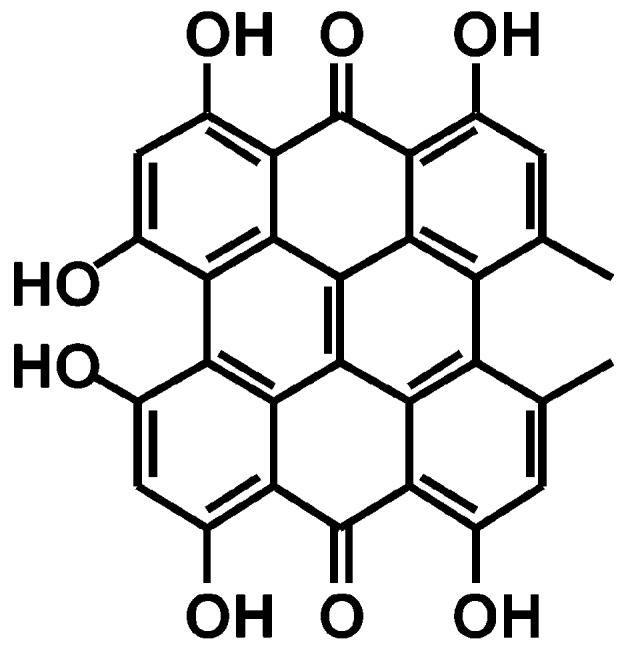
Chemical structure of hypericin.

Hypericin (Figure 1) is a common natural anthraquinone compound present in *Hypericum perforatum*, known as St. John’s wort, extracts [4,8,9,10]. Hypericin has been well known for years and exhibits antibacterial, antiviral, anticancer, and antidepressant activities [4,10,11,12]. This molecule is an established Ps that absorbs light in the visible range, especially at 590 nm in the orange region [5,13]. The hypericin structure contains both hydrophobic and hydrophilic portions that help it to penetrate the lipid bilayer of the bacterial membrane [14]. To generate enough ROS to kill bacteria, the Ps type, dose, and incubation time to bind the Ps to the cell must be considered [15,16], and hypericin enables both type 1 and type 2 photoactivation in the form of superoxide radicals [17].

In some cases, APDT has not caused efficient bacterial reduction, but only a very low log reduction. Indeed, one study reported that hypericin and orange light-treated *P. aeruginosa* exhibited only a 0.7 log reduction [18]. In another study, cell wall damage by APDT was observed by scanning electron microscopy in *S. aureus* cells, but not in *Escherichia coli* cells [15]. Nonetheless, this finding does not mean that *E. coli* cells are APDT resistant, and the main cause is probably related to the delivery of the Ps to the specific bacterial cells. Most previous studies have reported effective Gram-positive bacterial killing by APDT [4,15]. Gram-positive bacteria can take up anionic or neutral Ps due to their thick peptidoglycan layer on the outer surface [4,15], whereas Gram-negative bacteria fail to take up Ps because of the additional outer membrane and permeability barrier produced by lipopolysaccharides [19]. Thus, the Ps cannot penetrate well into the cell membrane, and after illumination, the effect of APDT may not be observed [19]. In contrast, APDT with the natural product hypericin has exhibited effective antimicrobial activity against Gram-positive bacteria (*S. aureus*) [10,15].

The yeast *Candida albicans* is often a transient colonizer on the mucous membranes of the mouth, throat, digestive tract, and skin [20]. However, *Candida* can cause mucosal lesions, skin infections, or serious and invasive systemic dissemination, especially in immunocompromised individuals. APDT with hypericin and visible light (602 ± 10 nm) effectively inactivates *C. albicans* [20].

*P. aeruginosa* is a well-known multidrug-resistant [21] pathogenic Gram-negative bacterium that is commonly found in water, soil, skin, and natural and artificial environments. *P. aeruginosa* is one of the most common pathogens that causes respiratory infections or nosocomial infections in hospitalized patients and is responsible for chronic lung infections [22]. This bacterium is able to develop resistance due to its unique property of changing its growth mode from a planktonic to a biofilm state [23]. Flagella and type 4 pili are important virulence factors, and *P. aeruginosa* has a type 3 secretion system (T3SS) that is responsible for toxin secretion and facilitates acute invasive infections, with high mortality [24,25,26]. *P. aeruginosa* is resistant to many common antibiotics, including ampicillin. β-Lactam antibiotics, such as ampicillin, penicillin, and cephalosporins, inhibit cell wall synthesis [27]; among different classes of β-lactamase enzymes, class D β-lactamases are oxacillin-hydrolyzing enzymes that are most common in Enterobacteriaceae and *P. aeruginosa* [28]. In a clinical study, urinary tract infection isolates were found to have 100% ampicillin resistance [21]. In another study with 97 clinical isolates of *Pseudomonas* strains, only 12.3% were found to be susceptible to ampicillin [29]. The unique properties of the *P. aeruginosa* outer membrane barrier interrupt the uptake of various antibiotics and chemicals [18,19]. The lack of effective killing of *P. aeruginosa* by hypericin APDT is probably because the outer membrane of *P. aeruginosa* prevents hypericin from entering the cell [18]. One possible way to overcome this problem is to increase the cell permeability by damaging the cell wall, and we speculate that after successful hypericin penetration, orange light exposure can induce enough ROS to kill the cells. In general, ROS accumulation is the direct or indirect cause of cell death [16].

Ampicillin may act as a penetration-facilitating agent to enable the effective use of hypericin as a Ps. However, as *P. aeruginosa* is already known to be resistant to ampicillin [30], this antibiotic cannot be used alone, but can be combined with hypericin for APDT. We hypothesized that ampicillin can attach easily to the cell membrane of Gram-negative cells and damage the cell membrane, thus increasing its permeability and Ps uptake. The combination of Ps (rose bengal and methylene blue) with antibiotics (murpirocin, linezolid) to kill Gram-positive *S. aureus* has been reported [31]. Furthermore, detergents and permeabilizing chemicals such as EDTA and CaCl_2_ have been suggested to enhance the APDT effect by increasing the membrane permeability of photosensitizers [19]. In the present study, we used hypericin (Ps) and ampicillin (a safe clinical antibiotic for damaging membrane permeability) to efficiently kill the Gram-negative bacterium *P. aeruginosa*.

*Caenorhabditis elegans* is a self-reproducing nematode that has been used for almost four decades in various fields of biology, and is a well-known animal model that has frequently been studied for bacterial pathogenesis, host immunity, and drug discovery, among others. This tiny worm, approximately 1 mm long, is transparent and able to produce 300 genetically identical worms within 3–4 days [32,33,34]. *C. elegans* has been reported as a model for Gram-positive and Gram-negative bacterial and fungal infections [35]. *P. aeruginosa* PAO1 was the first pathogen shown to infect and accumulate in the intestine of *C. elegans* [36], and this strain can also kill *C. elegans* [37]. In our study, we used the *C. elegans* model to demonstrate the effects of APDT on *P. aeruginosa* PAO1 pathogenesis through microscopic observation of worm growth and gut permeability. This study is the first to demonstrate the APDT method using hypericin and ampicillin cotreatment for *P. aeruginosa* inhibition and to evaluate these APDT effects in a *C. elegans* animal model in vivo.

## 2. Materials and Methods

### 2.1. Chemicals

Ampicillin sodium salt, dimethyl sulfoxide (DMSO), gentamicin, nystatin, and Brilliant Blue FCF were purchased from Sigma (St. Louis, MO, USA). Hypericin (molecular weight 504.44 g/mol) was purchased from Aktin Chemicals, Inc. (Chengdu, China), and its purity and chemical structure were checked by ^1^H-NMR spectroscopy and comparison with the previous literature, as seen in Figure S1 [38]. Hypericin was dissolved in DMSO for bacterial treatment. The stock solution was prepared at 50 mM, and the working solution was prepared at 10 µM. The stock solution was stored at −20 °C, and the working solution was kept at room temperature. The DMSO concentration was 0.1% for every sample, including the control. The ampicillin and gentamicin solution was prepared with autoclaved distilled water at a concentration of 100 mg/mL, and the final working concentration was 100 µg/mL. The nystatin stock was prepared with autoclaved distilled water at a concentration of 50 mg/mL, and the final working concentration was 20 µg/mL.

### 2.2. LED-Based Orange Light Source

The light source used for the experiment was an LED from Thorlabs (Newton, NJ, USA) emitting orange light at a wavelength of 590 nm. The intensity was measured by an LI-250A light meter (LI-COR Bioscience, Lincoln, NE, USA) in units of W/m^2^. The intensity of orange light used in this study was 150 ± 20 W/m^2^ at a distance of 1 cm from the LED.

### 2.3. Bacterial Cultures and Growth Conditions

*P. aeruginosa* PAO1, *S. aureus* KCTC 3881 and KCTC 1916, and *C. albicans* KCTC 7965 were obtained from the Korean Collections for Type Cultures (KCTC, Jeongeup, Korea), and methicillin-resistant *S. aureus* (MRSA) 2659 was isolated from a clinical specimen as described in a previous report [39]. These bacterial and fungal cells were grown in liquid Luria Bertani (LB) and Sabouraud dextrose (SB) broth, respectively, at 37 °C with shaking at 180 rpm overnight for 14–16 h to reach the stationary phase. The cultures were diluted with fresh LB and SB and then adjusted to OD values of 0.01–0.02 for *P. aeruginosa* PAO1, 0.05 for *S. aureus* and MRSA, and 0.03 for *C. albicans,* as measured with a spectrophotometer at a wavelength of 600 nm. *C. albicans* KCTC 7965 was cultured in SB broth overnight at 37 °C with shaking at 180 rpm. The initial cell numbers for *S. aureus*, MRSA, *P. aeruginosa*, and *C. albicans* were approximately 10^7^ to 10^8^ CFU/mL.

### 2.4. APDT of Bacteria and Fungi

#### 2.4.1. APDT of *S. aureus* and MRSA

Bacterial cultures of *S. aureus* KCTC 3881, KCTC 1916, and MRSA 2659 were incubated with hypericin (10 µM) for 30 min at room temperature in a shaker at 180 rpm and exposed to orange light for 1 h at 150 ± 20 W/m^2^. For light exposure, 100 µL samples were irradiated from the bottom of a transparent 96-well plate (SPL Life Science, Pocheon, Korea). The plate was covered with a lid during illumination to maintain sterile conditions. CFU/mL determination was performed using LB agar plates. Colonies were counted after 24 h at 37 °C. Ampicillin (100 µg/mL) was used as a positive control because this antibiotic effectively kills Gram-positive bacteria, such as *S. aureus* [40]. Triplicate agar plates were used for each CFU determination. Three independent experiments were performed.

#### 2.4.2. APDT of *C. albicans* KCTC 7965

A culture of *C. albicans* KCTC 7965 was incubated with hypericin (10 µM) for 30 min at room temperature in a shaker at 180 rpm and exposed to orange light for 1 h at 150 ± 20 W/m^2^. Nystatin (20 µg/mL) was used as a positive control. For light exposure, 100 µL samples were irradiated from the bottom of a transparent 96-well plate (SPL). The plate was covered with a lid during illumination to maintain sterile conditions. CFU/mL determination was performed using SB agar. Colonies were counted after 24–48 h at 37 °C. Triplicate agar plates were used for each CFU determination. Three independent experiments were performed.

#### 2.4.3. APDT of *P. aeruginosa* PAO1

A bacterial culture of *P. aeruginosa* PAO1 was incubated with hypericin (10 µM), ampicillin (100 µg/mL), or gentamicin (100 µg/mL) for 30 min at room temperature in a shaker at 180 rpm and exposed to orange light for 3 h at 150 ± 20 W/m^2^. Gentamicin (100 µg/mL) was used as a positive control. For light exposure, 100 µL samples were irradiated from the bottom of a transparent 96-well plate (SPL). The plate was covered with a lid during illumination to maintain sterile conditions. The *P. aeruginosa* PAO1 vehicle control (with only DMSO) was treated without hypericin and ampicillin. CFU/mL determination was performed using LB agar. Colonies were counted after 24 h at 37 °C. Triplicate agar plates were used for each CFU determination. Three independent experiments were performed.

### 2.5. Determination of Hypericin Intake in P. aeruginosa and S. aureus by Measuring the Fluorescence Intensity of Hypericin

An overnight culture of *P. aeruginosa* (0.03 OD value, 100 µL) was incubated with hypericin (10 µM), ampicillin (100 µg/mL), or both (10 µM hypericin and 100 µg/mL ampicillin) for 3.5 h in a shaker at 180 rpm at room temperature in a 96-well flat black cell culture plate (Corning Incorporated, Corning, NY, USA). The treated bacterial cells were centrifuged at 20,810× *g* for 30 min. The medium was carefully removed from each well, 100 µL of DPBS was added, and fluorescence images were obtained. For assessing *S. aureus* hypericin intake, an overnight culture (0.05 OD value, 100 µL) was treated with 10 µM hypericin for 30 min at room temperature, after which 100 µL of sample was transferred to a black 96-well plate (Corning) and incubated for 1 h; 50 µL of the supernatant was removed, and fluorescence images were obtained. The fluorescence images were captured with an Operetta system (excitation wavelength range of 460–490 nm and emission wavelength range of 560–630 nm; PerkinElmer, Waltham, MA, USA) and the image software Harmony version 3.5. The images were later analyzed by ImageJ software (version 1.4, National Institutes of Health, Bethesda, MD, USA).

### 2.6. C. elegans Strain and Maintenance

Wild-type *C. elegans* N2 and *E. coli* OP50 were obtained from the *Caenorhabditis* Genetics Center (CGC, Minneapolis, MN, USA). *C. elegans* was maintained on an NGM agar plate at 20 °C and fed live *E. coli* OP50. Age-synchronized eggs were prepared as described previously [41,42].

### 2.7. Toxicity Test of Orange Light on C. elegans

Age-synchronized eggs were incubated with *E. coli* OP50. At 24 h after egg preparation, young worms were exposed to orange light (LED, 590 nm wavelength, intensity 150 ± 20 W/m^2^) for 1 min, 10 min, 1 h, and 3 h to check the toxicity of orange light. The body length of the worms was measured at 96 h after egg preparation as described previously [41,42].

### 2.8. Evaluation of APDT in C. elegans by Measuring Growth Rate

APDT-treated *P. aeruginosa* PAO1 cells were fed to *C. elegans* from the egg stage. After 64 and 96 h of treatment, the body length was observed by using a stereoscopic zoom microscope (SMZ800N, Nikon, Tokyo, Japan) and a Progress Gryphax camera (Jenopitk, Germany). The body length of *C. elegans* was analyzed by ImageJ software as described previously [41,42].

### 2.9. Confirmation of APDT in C. elegans by Measuring Intestinal Permeability

The intestinal permeability of *C. elegans* was measured because bacterial toxicity can be clearly observed in this nematode model, especially in the case of *P. aeruginosa* PAO1-infected worms, where high toxicity leads to intestinal permeability dysfunction [36]. Age-synchronized L4 worms were treated with *E. coli* OP50, live *P. aeruginosa* PAO1, or APDT-treated *P. aeruginosa* PAO1 for 48 h at 20 °C. The treated worms were transferred to 96-well plates containing 100 µL of Brilliant Blue FCF and incubated for 3 h at 20 °C. The Brilliant Blue FCF was mixed with an *E. coli* OP50 culture to a concentration of 5% *w*/*v*. Worms treated with *E. coli* OP50 without Brilliant Blue FCF staining were used as a vehicle control. After staining, five to ten worms were transferred to microscope slides to observe the blue dye inside the intestines. Images were captured using a stereoscopic zoom microscope and a Progress Gryphax camera.

### 2.10. Statistics

The data are presented as the means ± SD. Statistical significance was determined by one-way analysis of variance (ANOVA) followed by Tukey’s multiple comparison test. The statistical software GraphPad Prism version 7.04 (La Jolla, CA, USA) was used to analyze the data and produce graphs. *p* value < 0.05 was considered statistically significant.

## 3. Results and Discussion

### 3.1. APDT Effectively Acts on the Gram-Positive Bacteria S. aureus and MRSA and the Fungus C. albicans

**Figure 2:**
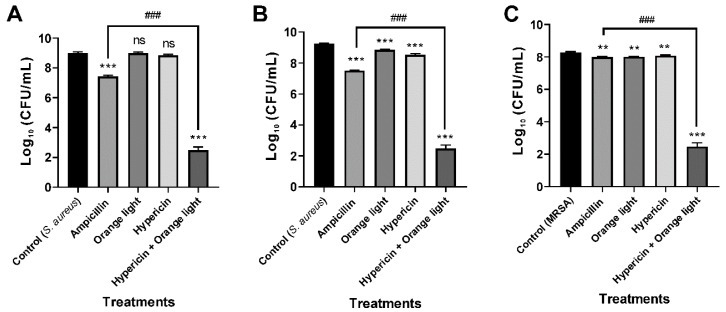
Antimicrobial photodynamic therapy (APDT) for Gram-positive bacteria, *S. aureus* and methicillin-resistant *Staphylococcus aureus* (MRSA). APDT with hypericin and orange light in *S. aureus* KCTC 3881 and KCTC 1916 (**A**,**B**) and the methicillin-resistant strain (MRSA) 2659 (**C**). An overnight culture of bacteria was treated with hypericin (10 µM) and ampicillin (100 µg/mL, a positive control) in a shaking incubator for 30 min. Then, the bacterial sample was exposed to orange light with an intensity of 150 ± 20 W/m^2^ from underneath for 1 h in a transparent 96-well plate. Each value corresponds to the mean ± standard deviation from triplicate experiments. The graphs are representatives of three independent experiments. ** *p* < 0.01 and *** *p* < 0.001, difference from the vehicle control; ns, no significant difference from the vehicle control; ^###^ *p* < 0.001, difference from the ampicillin single treatment.

First, we evaluated APDT on the Gram-positive bacterium *S. aureus* to test the suitability of our APDT setup. Ampicillin (100 µg/mL) was used as a positive control. The optimum APDT setup was hypericin (10 µM) pretreatment for 30 min followed by orange light for 1 h. The data show that the combined treatment of hypericin with orange light significantly decreased the growth of *S. aureus* KCTC 3881, KCTC 1916, and MRSA 2659 compared to that of the vehicle control (*p* < 0.001). Moreover, the APDT effect was significantly higher than that of the conventional ampicillin treatment against *S. aureus* KCTC 3881 (*p* < 0.001), KCTC 1916 (*p* < 0.001), and MRSA 2659 (*p* < 0.001), as seen in Figure 2. These data are consistent with previous reports of APDT against the Gram-positive bacterium *S. aureus* [18].

**Figure 3:**
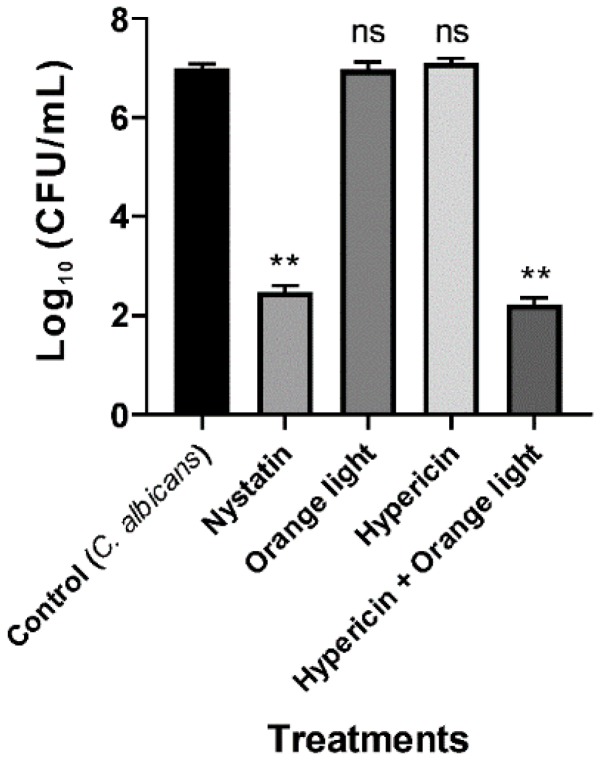
APDT of *C. albicans* KCTC 7965 with hypericin and orange light. Overnight culture of *C. albicans* at 37 °C in SB broth. Cells were incubated with hypericin (10 µM) or nystatin (20 µg/mL, a positive control) at room temperature in a shaking incubator for 30 min. Then, the fungal sample was exposed to orange light with an intensity of 150 ± 20 W/m^2^ from underneath for 1 h in a transparent 96-well plate. Each value corresponds to the mean ± standard deviation from triplicate experiments. The graph is a representative of three independent experiments. ** *p* < 0.01, difference from the vehicle control. ns—no significant difference from the vehicle control.

We next tested APDT on the fungal pathogen *C. albicans*, and the data showed that the combined treatment effectively inhibited growth, which was consistent with previous reports [20,43]. *C. albicans* KCTC 7965 was treated with hypericin (10 µM) for 30 min followed by orange light for 1 h. Nystatin (20 µg/mL) was used as a positive control. APDT also inhibited fungal growth (*p* < 0.01) and caused a 4.8 log reduction, as seen in Figure 3.

In this study, we used an incubation time of 30 min to allow sufficient binding of hypericin to the bacterial and fungal cells. In the case of light treatment, the light-emitting diode (LED) source was convenient for application in the APDT system because of its easy universal handling features. The main advantages of using an LED are resistance, durability, wavelength selectivity, low heat emission, and lower cost than other conventional light sources [18,44].

### 3.2. Conventional APDT with Hypericin and Orange Light Did Not Kill P. aeruginosa PAO1

**Figure 4:**
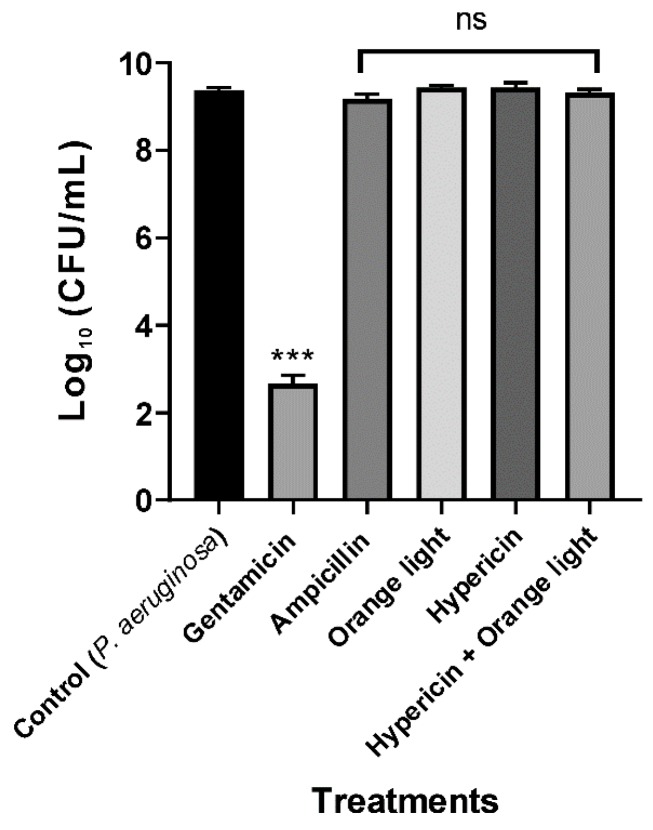
APDT of *P. aeruginosa* PAO1 with hypericin and orange light. An overnight culture was incubated with ampicillin (100 µg/mL), hypericin (10 µM), or gentamicin (100 µg/mL, a positive control) for 30 min in a shaking incubator at room temperature. Then, the bacterial sample was exposed to orange light with an intensity of 150 ± 20 W/m^2^ from underneath for 3 h in a transparent 96-well plate. Each value corresponds to the mean ± standard deviation from triplicate experiments. The graph is a representative of three independent assays. *** *p <* 0.001, difference from the vehicle control. No significance (ns) was found for any of the treatments compared with the vehicle control.

*P. aeruginosa* ATCC 27853 was not efficiently killed by hypericin and light APDT in a previous study [18]. In the present study, we found similar results for the treatment of *P. aeruginosa* PAO1 with hypericin for 30 min at room temperature and orange light for up to 3 h, as seen in Figure 4, even though this treatment worked well for killing *S. aureus*, as seen in Figure 2. We hypothesized that the different APDT effects against Gram-positive and Gram-negative bacteria are probably due to differences in hypericin membrane permeability.

### 3.3. Hypericin and Ampicillin Cotreatment Followed by Orange Light Significantly Inhibited P. aeruginosa PAO1

**Figure 5:**
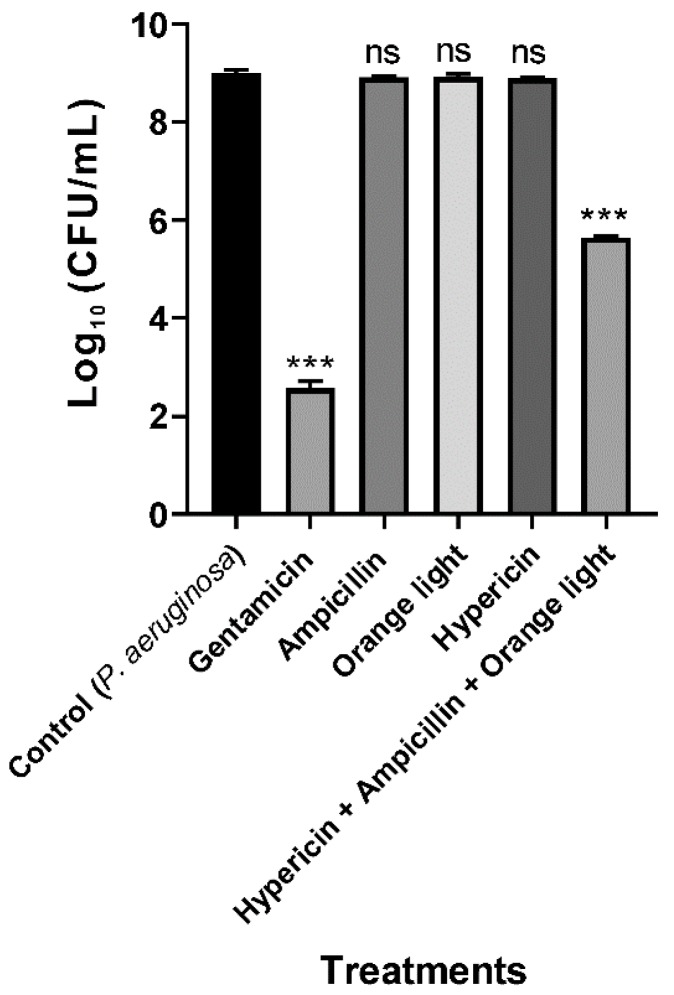
APDT with hypericin and ampicillin cotreatment followed by orange light for the Gram-negative bacterium *P. aeruginosa* PAO1. An overnight culture was incubated with gentamicin (100 µg/mL, a positive control), ampicillin (100 µg/mL), hypericin (10 µM), or hypericin and ampicillin cotreatment for 30 min in a shaking incubator at room temperature. Then, the bacterial sample was exposed to orange light with an intensity of 150 ± 20 W/m^2^ from underneath for 3 h in a transparent 96-well plate. Each value corresponds to the mean ± standard deviation of triplicate experiments. The graph is a representative of three independent experiments. *** *p* < 0.001, difference from the vehicle control. ns—no significant difference from the vehicle control.

To increase the efficiency of the hypericin APDT, we used ampicillin cotreatment to damage the cell walls and thereby increase hypericin uptake into the cell. According to the USA Food and Drug Administration’s Tentative Final Monograph for Healthcare Antiseptic Drug Products, bacterial inhibition or reduction by any treatment should be a minimum of 3 log to constitute a good antibacterial effect [45]. In our combination APDT treatment, we achieved a maximum 3.4 log reduction, which indicates that this strategy is successful for killing *P. aeruginosa* PAO1, as seen in Figure 5. Ampicillin alone or hypericin alone did not have an effective log reduction with the same treatment time and method. We believe that this result is due to a synergistic effect of hypericin and ampicillin. In addition, we can assume that a new conjugate molecule combining a Ps and an antibiotic together can act as a new and effective APDT agent.

### 3.4. Ampicillin Facilitated Hypericin Uptake in the Gram-Negative Bacterium P. aeruginosa PAO1

**Figure 6:**
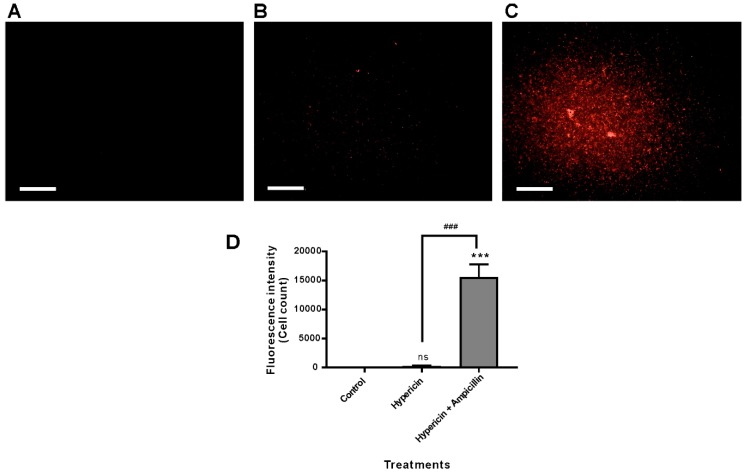
Hypericin uptake in *P. aeruginosa* PAO1 bacterial cells was analyzed using fluorescence microscopy imaging and quantification. *P. aeruginosa* PAO1 was treated with hypericin (10 µM) or cotreated with hypericin (10 µM) and ampicillin (100 µg/mL) for 3.5 h, and fluorescence microscopy images were obtained using the Operetta system. (**A**) Vehicle control; (**B**) hypericin 10 µM; (**C**) cotreatment with 10 µM hypericin and 100 µg/mL ampicillin. Scale bar = 100 µm. (**D**) Quantification of hypericin fluorescence in bacterial cells. Each value corresponds to the mean ± standard deviation from triplicate experiments. The data are representative of at least three independent assays. *** *p* < 0.001, difference from the vehicle control; ns, no significant difference from the vehicle control. ^###^ *p* < 0.001, comparison between the hypericin single treatment and cotreatment with ampicillin and hypericin.

Our hypothesis was that ampicillin may damage the membrane of the Gram-negative bacterium *P. aeruginosa* PAO1 and facilitate hypericin penetration and uptake. To test this hypothesis, we observed the fluorescence intensity of hypericin in different bacterial cells. Hypericin alone exhibited very low fluorescence in *P. aeruginosa* PAO1 cells, which was clearly observed by fluorescence measurement in Operetta imaging, which can be interpreted in relation to images taken after treatment with hypericin and ampicillin, as seen in Figure 6. Although hypericin alone had low intensity, 10 µM hypericin and 100 µg/mL ampicillin together resulted in a very strong and clear signal of hypericin in the bacterial cells. Significantly higher hypericin fluorescence intensity was observed with hypericin and ampicillin cotreatment than the vehicle control and the hypericin single treatment (*p* < 0.001), as seen in Figure 6A–D.

**Figure 7:**
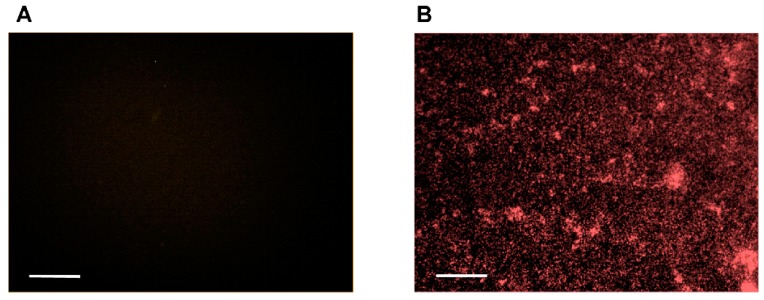
Hypericin uptake by *S. aureus* bacterial cells was analyzed using fluorescence microscopy imaging and quantification. *S. aureus* was incubated with (**A**) the vehicle control and (**B**) hypericin (10 µM) for 30 min, and fluorescence microscopy images were obtained using the Operetta system. Scale bar = 100 µm. The data are representative of two independent assays.

In addition, as expected, hypericin uptake by *S. aureus* resulted in a very strong fluorescence signal after 30 min of incubation with hypericin, as seen in Figure 7. Based on the data, we speculate again that the different effects of APDT against Gram-positive (*S. aureus*) and Gram-negative bacteria (*P. aeruginosa*) originated from differences in membrane permeability to hypericin.

### 3.5. APDT with Ampicillin and Hypericin Was Evaluated in the C. elegans Model by Measuring Worm Growth

**Figure 8:**
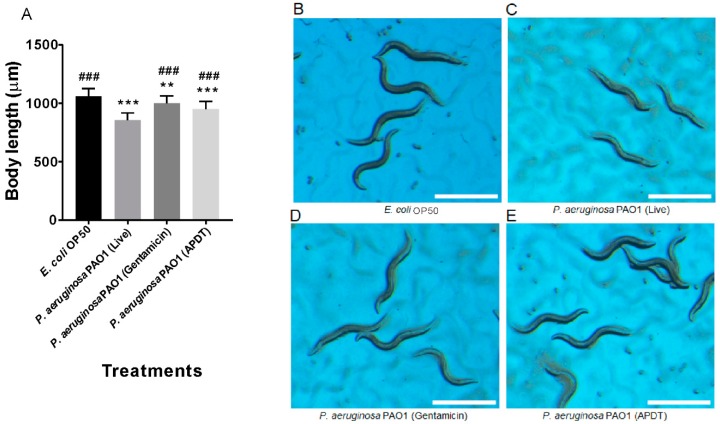
Pathogenesis evaluation of *P. aeruginosa* PAO1 in *C. elegans* by measurement of worm growth at 96 h. Body length measurement graph (**A**). Age-synchronized *C. elegans* eggs were fed the vehicle control *E. coli* OP50 (**B**), live *P. aeruginosa* PAO1 (**C**), gentamicin (100 µg/mL)-treated *P. aeruginosa* (**D**), and APDT-treated *P. aeruginosa* (**E**) for 96 h. Scale bar = 1 mm. Each value corresponds to the mean ± standard deviation (*n* = 30). ** *p* < 0.01 and *** *p* < 0.001, difference from the vehicle control. ^###^ *p* < 0.001, difference from the live *P. aeruginosa*-fed worms.

*P. aeruginosa* is a good pathogen model for severe infection in *C. elegans* [36], which is itself a representative animal model for observing the therapeutic effects and side effects of biological treatments. It has already been reported that *P. aeruginosa* PAO1 is toxic and pathogenic to *C. elegans*, and can rapidly infect these nematodes [36]. For all treatments, including the control, bacterial cells were fed to the egg stage of N2 wildtype *C. elegans*, and the growth rate was evaluated by measuring body length after 96 h, as seen in Figure 8. Worms fed live *P. aeruginosa* PAO1 showed significantly smaller body lengths than vehicle control worms (*p* < 0.001), as seen in Figure 8A–C. The gentamicin- and APDT-treated groups displayed significantly increased body length compared to that of the live *P. aeruginosa* treatment group (*p* < 0.001), as seen in Figure 8A,C–E. Similarly, after 64 h of treatment, the live *P. aeruginosa* PAO1-fed worms exhibited significantly smaller body sizes than the vehicle control-treated worms (*p* < 0.001). In contrast, APDT- or gentamicin-treated *P. aeruginosa*-fed worms had body sizes similar to those of the vehicle control-treated worms, as seen in Figure S2. Based on these data, we conclude that APDT can be successfully applied to the animal model *C. elegans* without apparent side effects.

### 3.6. APDT Rescued Intestinal Permeability Dysfunction in C. elegans

**Figure 9:**
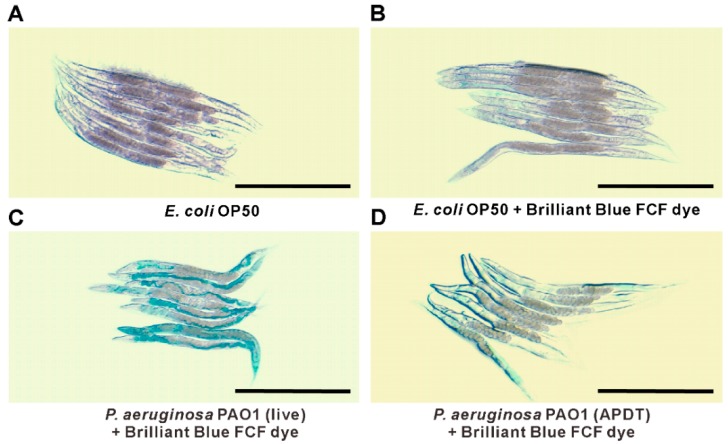
*P. aeruginosa* PAO1 pathogenicity observation by gut permeability with Brilliant Blue FCF assay. Stereomicroscope images of worms treated with *E. coli* OP50 without Brilliant Blue FCF dye (**A**), *E. coli* OP50 with Brilliant Blue FCF dye (**B**), live *P. aeruginosa* PAO1 with Brilliant Blue FCF dye (**C**), and APDT-treated *P. aeruginosa* with Brilliant Blue FCF dye (**D**). Scale bar = 500 µm. Age-synchronized L4 worms were fed *E. coli* OP50 (**A**,**B**), live *P. aeruginosa* PAO1 (**C**), or APDT-treated *P. aeruginosa* for 48 h at 20 °C. Then, the Brilliant Blue FCF dye was fed to the worms for 3 h at 20 °C (**B**–**D**), except for the vehicle control consisting of worms fed *E. coli* without dye (**A**). The microscope images are representative of three independent experiments.

*P. aeruginosa* PAO1 can easily cause serious intestinal infection within a very short time in *C. elegans* [36]. To clearly understand the infection model, we used Brilliant Blue FCF dye, which facilitates the evaluation of gut permeability during infection by *P. aeruginosa* PAO1. We found a clear difference based on microscopic observation after *C. elegans* was fed APDT-treated and live *P. aeruginosa* PAO1 for 48 h. After 3 h of staining, we observed that most infected worms absorbed the dye, as their gut was damaged, with deep blue staining, as seen in Figure 9C. Conversely, APDT *P. aeruginosa*-fed worms showed little staining or blue color in the body, indicating that their gut was not as damaged and that they were in good condition, as seen in Figure 9D. The *E. coli* OP50 strain was used as a vehicle control, and *E. coli*-fed worms displayed little dye in the intestine, with no leakage found, as seen in Figure 9B. Consequently, this APDT approach can be a good treatment method for *P. aeruginosa* intestinal infection.

We can administer hypericin and ampicillin together, followed by orange light treatment, and may achieve good bactericidal effects. Ampicillin alone cannot kill *P. aeruginosa* PAO1 completely or significantly, but this APDT method can be readily applied in the future with other Gram-negative bacteria in identical approaches. APDT with hypericin, ampicillin, and orange light showed promise in this study. Pathogenesis evaluation by the Brilliant Blue FCF assay for gut permeability in the *C. elegans* system, as we describe herein, is a unique method for testing APDT effects.

Photodynamic treatment with orange light and the Ps hypericin was reported to successfully decrease tumor volume in the nude mouse xenograft model, and orange light laser (589 nm) alone did not cause significant skin injury [46]. Similarly, in this study, we checked the harmful effect of an orange LED at a wavelength of 590 nm in advance, as seen in Figure S3. In biophotonic studies, it is easy to observe the toxic effect in the *C. elegans* system with a simple experiment by measuring their growth rate or survival [36,41]. In our experiment, we treated worms with the same intensity of orange light (150 ± 20 W/m^2^) used for bacterial inactivation for 1 min to 3 h and found no significant damage from orange light compared to that in the vehicle control, as seen in Figure S3.

Mouse and rat animal models are used as in vivo models for the treatment of bacterial infections and tumors to observe and compare the improvement resulting from PDT [46,47,48]. In this study, we used the model nematode *C. elegans*. Compared to mammalian animal systems, this nematode system is convenient and cost-effective, with fewer ethical issues [41,42]. Thus, by examining the growth rate and gut permeability in worms, we demonstrate for the first time that APDT with hypericin, ampicillin, and orange light has less toxicity than live *P. aeruginosa* PAO1. *C. elegans* are tiny nematodes, are easy to cultivate, and require a short amount of time for any experimental setup, as their lifespan is short, i.e., approximately 3 weeks [32]. We have established a model for determining the effectiveness of APDT by establishing CFU/mL as a conventional method as well as an in vivo efficacy test using the *C. elegans* system by measuring growth rate and gut permeability. This approach is a complete testing procedure for any APDT experiment, including both in vitro and in vivo analyses.

APDT is a very promising, effective, low-cost, easily applicable, and environmentally safe method. It can be applied in various fields, for example, hospitals, food industries, or wastewater treatment. The penetration depth and efficiency of light are critical, especially in clinical PDT applications [49], and orange light (wavelength 590–620 nm) is known to penetrate approximately 1.5 mm into tissue [50]. We are also screening new Ps candidates from natural products that can react with red light (wavelength 620–750 nm), which has an effective penetration depth of up to 3 mm [50].

**Figure 10:**
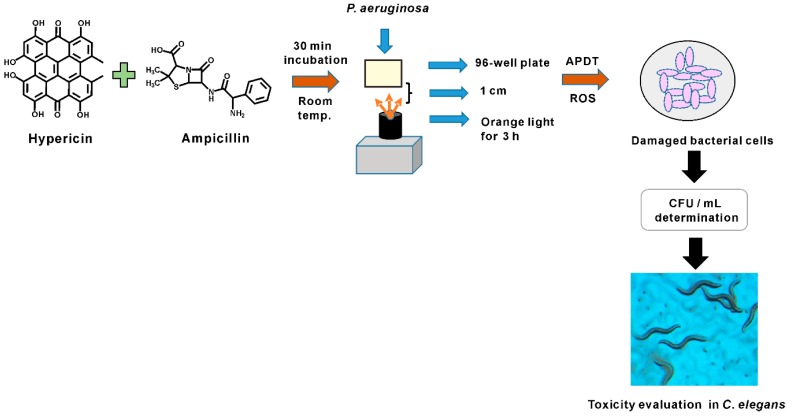
Summary figure for *P. aeruginosa* PAO1 APDT treatment experimental setup.

In this study, successful APDT for *P. aeruginosa* PAO1 bacterial cells was achieved by hypericin, ampicillin, and orange light treatment. *P. aeruginosa* has 12–100 times lower outer membrane permeability than *E. coli* [18]. Therefore, a challenge of hypericin treatment is to move enough hypericin across the outer membrane to achieve a significant APDT effect. This difficulty is the main reason for the failure of current hypericin APDT for *P. aeruginosa*. To address this issue, we established a hypericin uptake mechanism by fluorescence imaging of *P. aeruginosa* PAO1 cells treated with hypericin alone and in combination with ampicillin. Our hypothesis was that ampicillin may increase cell permeability, allowing hypericin to easily penetrate the cell. Accordingly, we observed that hypericin treatment alone produced very low fluorescence compared to that produced by ampicillin and hypericin cotreatment. We thus showed that ampicillin can act as an agent for successful transport of hypericin. This method was established for the first time and resulted in effective APDT against *P. aeruginosa* PAO1, probably via the mechanisms proposed. After the in vitro APDT assessment, efficacy and toxicity were evaluated in an in vivo nematode model system, revealing that our APDT treatment produced sufficient bacterial inhibition with orange light exposure for 3 h. This strategy is an excellent method, as APDT-treated *P. aeruginosa*-fed worms experienced lower toxicity than worms fed live *P. aeruginosa*. The experimental setup and the process are briefly summarized in Figure 10.

## 4. Conclusions

Killing Gram-negative bacteria by APDT is difficult due to differences in their cellular membrane structures from those of Gram-positive bacteria. *P. aeruginosa* is a multidrug-resistant pathogenic bacterium that is difficult to kill by the use of natural Ps, such as hypericin and orange light treatment. *P. aeruginosa* PAO1, an ampicillin-resistant strain, was successfully inhibited by up to 3.4 log by hypericin and ampicillin cotreatment followed by 3 h orange light treatment. The mechanism by which ampicillin treatment increased hypericin uptake was described, and the efficacy of APDT was evaluated in the *C. elegans* system. This new and unique strategy of Gram-negative APDT can serve as a model for killing other antibiotic-resistant bacteria in the future.

## Supplementary Materials

The following are available online at https://www.mdpi.com/1999-4923/11/12/641/s1, Figure S1: ^1^H-NMR spectrum of hypericin used in this study. Figure S2: Pathogenesis assessment for *P. aeruginosa* PAO1 in *C. elegans* by measurement of worm growth at 64 h. Figure S3: Toxicity test of orange light exposure in *C. elegans* fed *E. coli* OP50.
