# Supplementary material for: Antimicrobial Biophotonic Treatment of Ampicillin-Resistant Pseudomonas aeruginosa with Hypericin and Ampicillin Cotreatment Followed by Orange Light
Source: Pharmaceutics. 2019 Dec 1;11(12):641. doi: 10.3390/pharmaceutics11120641 (PMC6956302; doi:10.3390/pharmaceutics11120641)
Supplement: Supplementary file 1 [file pharmaceutics-11-00641-s001.pdf]

pharmaceutics

MDPI

Supplementary Materials: Antimicrobial Biophotonic Treatment of Ampicillin-Resistant *Pseudomonas aeruginosa* with Hypericin and Ampicillin Cotreatment Followed by Orange Light

Seemi Tasnim Alam, Tram Anh Ngoc Le, Jin-Soo Park, Hak Cheol Kwon and Kyungsu Kang

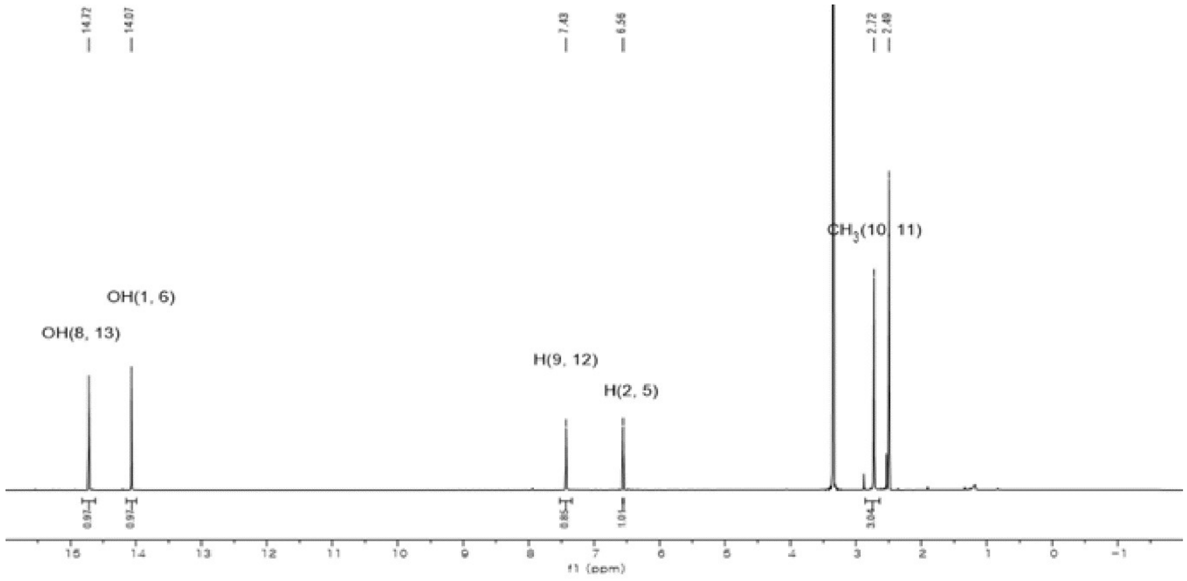

Figure S1. ^{1}H-NMR spectrum of hypericin used in this study (in DMSO-d_6).

^{1}H NMR (500 MHz, DMSO-d_6) δ 14.72 (s, 1H), 14.07 (s, 1H), 7.43 (s, 1H), 6.56 (d, J = 1.0 Hz, 1H), 2.72 (s, 3H).

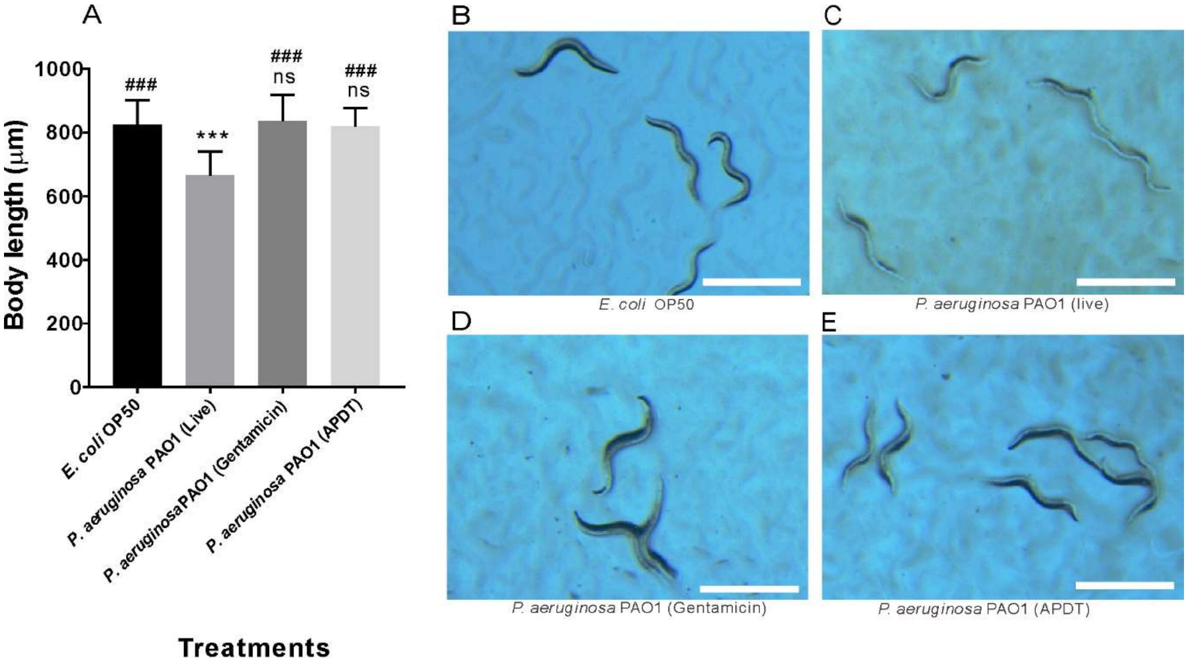

Figure S2. Pathogenesis assessment for *P. aeruginosa* PAO1 in *C. elegans* by measurement of worm growth at 64 h. Body length measurement graph (A). Age-synchronized *C. elegans* eggs were treated with the control, *E. coli* OP50 (B), live *P. aeruginosa* PAO1 (C), gentamicin (100 μg/mL)-treated *P. aeruginosa* (D), or APDT-treated *P. aeruginosa* (E) for 64 h. Scale bar = 1 mm. Each value corresponds to the mean ± standard deviation (n = 30).

*** $p < 0.001$, difference from the vehicle control.

$p < 0.001$, difference from the live *P. aeruginosa* treatment group.

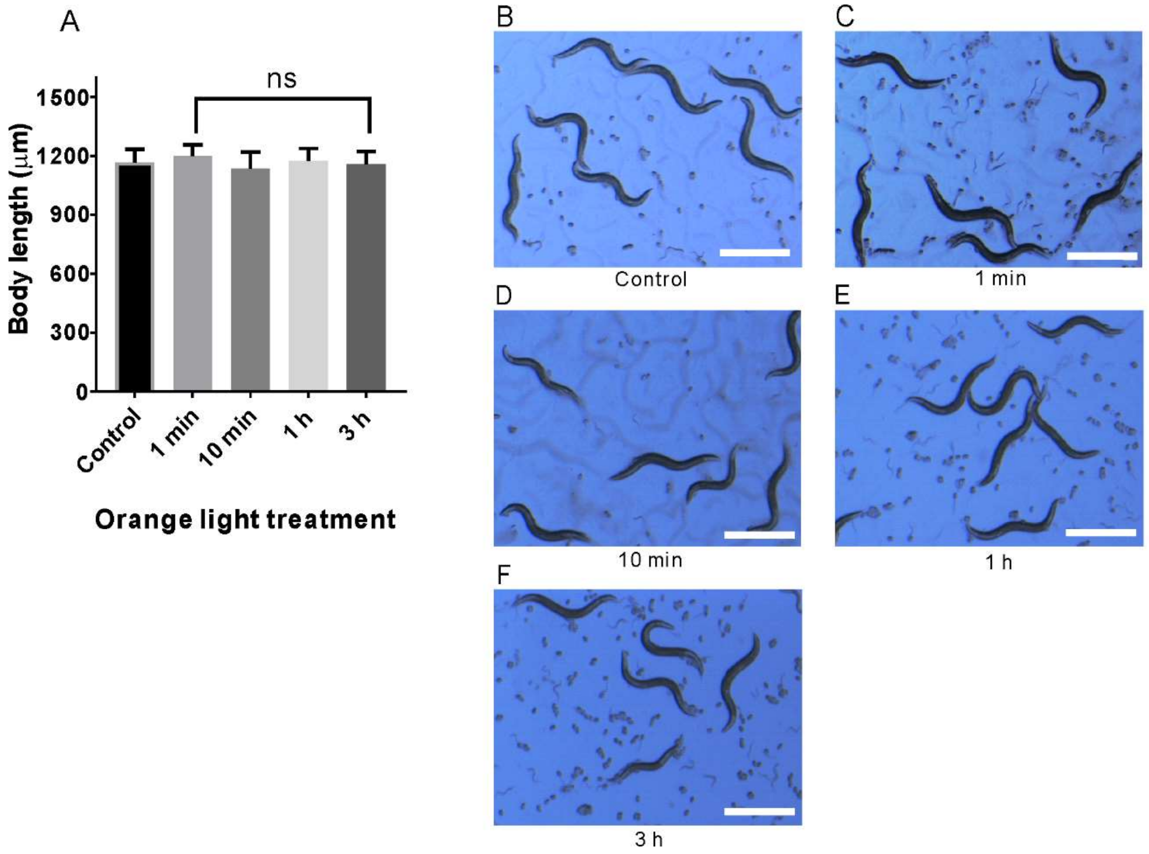

Figure S3. Toxicity test of orange light exposure in *C. elegans* fed *E. coli* OP50. Age-synchronized L1-stage worms were treated with orange light (150 ± 20 W/m^2) for 1 min, 10 min, 1 h, and 3 h. Then, the body length of worms was measured at 96 h after the egg preparation. The graph is representative of two independent experiments. Scale bar = 1 mm. No significant difference (ns) observed compared with the vehicle control (n = 50).
